# FABP4 as an immunometabolic hub in preeclampsia: from maternal-fetal interface to systemic inflammation

**DOI:** 10.3389/fimmu.2026.1856187

**Published:** 2026-06-12

**Authors:** Hui Sun, Fei Gu, Lipei Wu, Xin Chen, Shihai Xuan

**Affiliations:** Medical Laboratory Department, Affiliated Dongtai Hospital of Nantong University, Dongtai, Jiangsu, China

**Keywords:** endothelial dysfunction, FABP4, immunometabolism, macrophage polarization, maternal-fetal interface, preeclampsia

## Abstract

Preeclampsia (PE) is a multisystem vascular disease that occurs specifically during pregnancy, and the understanding of its pathogenesis is gradually shifting from the traditional “placental ischemia-endothelial injury” model to immunometabolic disorders. In this article, we propose a conceptual framework positioning fatty acid binding protein 4 (FABP4) as an immunometabolic hub in the pathogenesis of PE. Clinical studies suggest that FABP4 is significantly elevated in preeclamptic placentas and maternal circulation; epigenetic derepression via miR-148a/152-mediated DNMT1 downregulation contributes to this placental upregulation. Leveraging its primary identity as an intracellular lipid chaperone, we hypothesized a spatiotemporal cascade linking local immune imbalance to systemic vascular injury. Intracellularly, drawing upon highly conserved lipotoxic pathways established in non-pregnancy models, we extrapolate that FABP4 participates in the regulation of the immune microenvironment at the maternal-fetal interface by modulating macrophage polarization toward the M1-type and activating the NLRP3 inflammasome axis; a recent study in an EVT-derived cell line (HTR-8/SVneo) demonstrated that pharmacological inhibition or siRNA knockdown of FABP4 impairs mitochondrial membrane potential, reduces ATP synthesis, and increases oxidative stress, resulting in proliferative arrest. These findings position FABP4 as a viability factor for trophoblast-like cells under metabolic stress, though direct validation in primary EVTs is still required. Extracellularly, upon entering maternal circulation, FABP4 may trigger systemic inflammatory cascade responses and induce endothelial dysfunction. While single-cell transcriptomic studies have revealed significant reprogramming of lipid metabolism-related gene expression in PE placental immune cells, direct single-cell quantification of FABP4 across specific placental subpopulations remains to be performed. Consequently, by integrating macroscopic clinical data with these microscopic intercellular networks, we frame the FABP4-driven axis as a plausible mechanistic convergence, highlighting its promise as a critical translational target for immunometabolic intervention.

## 1 Introduction

### 1.1 Immunometabolic nature of preeclampsia

Preeclampsia (PE) is a new-onset multisystemic disorder of hypertension with proteinuria after 20 weeks of gestation, which is clinically categorized into early (<34 weeks) and late (≥34 weeks) forms (1). Its global prevalence is approximately 2–8%, and it is one of the leading causes of maternal and perinatal morbidity and mortality (2, 3). Although the clinical use of the soluble fms-like tyrosine kinase-1 (sFlt-1)/placental growth factor (PlGF) ratio has improved diagnostic accuracy (4), the “placental ischemia” reflected by these markers is merely a downstream consequence of disease progression and not necessarily its core cause, and the pathogenesis of the disease prior to the onset of clinical symptoms remains unclear. The traditional “placental ischemia-endothelial injury” model interprets PE as a vascular lesion secondary to inadequate perfusion (5). However, clinical observations indicate that subclinical low-grade inflammation precedes disease onset in some patients, indicating a self-amplifying relationship between inflammation and ischemic injury.

Recent Mendelian randomization studies support a causal link between specific lipid components and PE risk (6, 7), suggesting that dyslipidemia is an upstream driver of PE rather than a secondary consequence of placental injury (6). However, how dyslipidemia translates into a pathologic phenotype is unclear. Emerging insights in immunometabolism have pointed to intracellular metabolic pathways that are involved not only in energy supply but also modulate immune cell function through intermediate metabolites (8, 9). For example, tricarboxylic acid cycle metabolites (e.g., succinic acid and itaconic acid) can directly modulate macrophage polarization status (10), suggesting that metabolic disorders participate in inflammatory responses by reprogramming immune cells.

In the pathological process of PE, maternal metabolic syndrome, characterized by insulin resistance, obesity, and hyperlipidemia, acts synergistically with localized placental disorders of lipid metabolism, which abnormally activate macrophages at the maternal-fetal interface and disrupt the pre-existing state of immune tolerance (8, 11), and participates in the pathological process of impaired placental vascular remodeling and systemic endothelial injury.

### 1.2 Transformation of FABP4 from a lipid chaperone to an immunometabolic hub

Fatty acid binding protein 4 (FABP4) is an important molecule involved in the regulation of lipid metabolism. Previous studies have shown that FABP4 is the major intracellular lipid chaperone in adipocytes, being mainly responsible for transporting long-chain fatty acids, coordinating lipid storage, and maintaining systemic insulin sensitivity (12). Makowski et al. found that knockdown of FABP4 in macrophages attenuated atherosclerotic lesions (13), suggesting that this molecule is involved in metabolic inflammation. Subsequent studies have primarily focused on the metabolic aspects of obesity and cardiovascular diseases (11, 14).

Research progress suggests that FABP4 functions as an immunometabolic hub and is also a secreted adipokine; however, its secretory form and extracellular mechanism of action remain to be elucidated (14). In addition to adipocytes, FABP4 is expressed in immune cells, such as macrophages and dendritic cells (15, 16), and its expression level can be upregulated under metabolic stress (17).

Intracellular FABP4 is involved in the metabolic reprogramming of immune cells. Specifically, FABP4 expression is associated with pro-inflammatory M1-type macrophage polarization (18, 19)and participates in innate immune regulation by modulating mitochondrial function with inflammasome assembly and influencing NOD-like receptor family pyrin domain containing 3(NLRP3)inflammasome activation (19, 20). *In vitro*, FABP4 inhibitors modulate the NF-κB and PPARγ signaling pathways and attenuate lipopolysaccharide-induced endothelial cell injury (21).

In addition to its intracellular effects, FABP4, which enters the circulatory system, amplifies systemic inflammation. It acts directly on vascular endothelial cells, upregulates the expression of adhesion molecules, and promotes peripheral monocyte infiltration (22, 23). Notably, in pathological states, such as PE, FABP4 has been reported to form complexes or share downstream pathways with danger-associated molecular patterns, such as high mobility group box 1 (HMGB1), which together drive the M1-type polarization of macrophages at the maternal-fetal interface, thereby destroying the immune tolerance system (24), underscoring its immunomodulatory role. FABP4 thus operates as both an intracellular metabolic regulator and an extracellular immunostimulatory factor, bridging lipid metabolism and inflammatory responses. This positioning connects systemic metabolic disturbances with local immune dysregulation at the maternal-fetal interface.

Therefore, the specific rationale for isolating FABP4 as a primary focal point, among the myriad of proteins related to inflammatory and metabolic stress, lies in its unique temporal and cellular position in disease progression. Classical biomarkers such as the sFlt-1/PlGF ratio primarily reflect downstream endothelial injury and established placental ischemia (25), serving as secondary consequences rather than early warning signals. Similarly, ubiquitous pro-inflammatory cytokines represent late-stage systemic responses that lack predictive specificity for PE onset. In contrast, prospective clinical cohorts have demonstrated that maternal serum FABP4 levels are abnormally elevated as early as the first trimester (26), long before the clinical onset of hypertension and proteinuria, positioning it as an upstream sentinel of immunometabolic dysregulation rather than a late-stage byproduct. Moreover, unlike tissue-specific homologs such as FABP1 (liver-restricted) or FABP3 (muscle/heart-restricted), which lack validated pregnancy-specific predictive utility (27), FABP4 is uniquely co-expressed in both adipose tissue (14) and placental immune cells (28), enabling it to physically couple systemic lipid flux to local innate immune reprogramming. Investigating FABP4 is therefore particularly valuable because it provides a rare diagnostic and therapeutic window into the subclinical phase of PE, especially in patients with metabolic comorbidities before irreversible vascular damage occurs.

### 1.3 Scope and objectives of the study

The role of FABP4 in metabolic syndrome is well documented, but its regulation within the specific immune landscape of the maternal-fetal interface, particularly during PE, is far less clear. While previous reviews have addressed FABP4 in the context of reproductive biology and offspring metabolic outcomes (29) or general maternal-fetal health (30), the precise cellular drivers linking FABP4 to pregnancy-specific immunopathology, placental ischemia, and subsequent vascular injury remain underexplored (31, 32).

Recent advances in single-cell and single-nucleus RNA sequencing (33–35), combined with spatial transcriptomics (36), have opened a window into the molecular intricacies of the PE placenta. In this review, we summarize the current picture of FABP4 signaling within distinct immune populations, chart how pathological signals propagate from the placental bed into the maternal bloodstream, and assess whether FABP4 holds real weight as a future biomarker or therapeutic target in PE.

Rather than surveying metabolic regulation broadly, we have focused specifically on FABP4-mediated PE progression, emphasizing the transition from localized inflammation at the maternal-fetal interface to systemic vascular dysfunction and highlighting immune cell-specific mechanisms.

## 2 Intracellular mechanisms of FABP4 in immune cells

### 2.1 Expression profiles and phenotypic regulation in immune cells

Although traditionally regarded as a lipid chaperone protein, FABP4 is highly expressed in the immune system and is involved in lipid-induced inflammatory responses (37, 38). In myeloid cells, macrophages express higher levels of FABP4, which can be dynamically upregulated by metabolic stressors such as lipopolysaccharide (LPS) and free fatty acids (15, 18). Studies have shown that FABP4 expression levels correlate with macrophage polarization status; knockdown or pharmacological inhibition suppresses the proinflammatory M1 phenotype and promotes the expression of M2-type-related markers (39, 40), indicating FABP4 as a candidate target for modulating metabolic inflammation (41), although its therapeutic relevance in PE remains to be tested.

These intracellular mechanisms are currently established mainly in non-pregnancy metabolic disease models (e.g., metabolic syndrome, atherosclerosis, diabetic nephropathy) and should be interpreted as mechanistic analogies rather than direct equivalents of PE pathogenesis. Their applicability at the maternal-fetal interface of PE remains to be validated.

### 2.2 Molecular mechanisms of immunometabolic reprogramming

**Figure 1:**
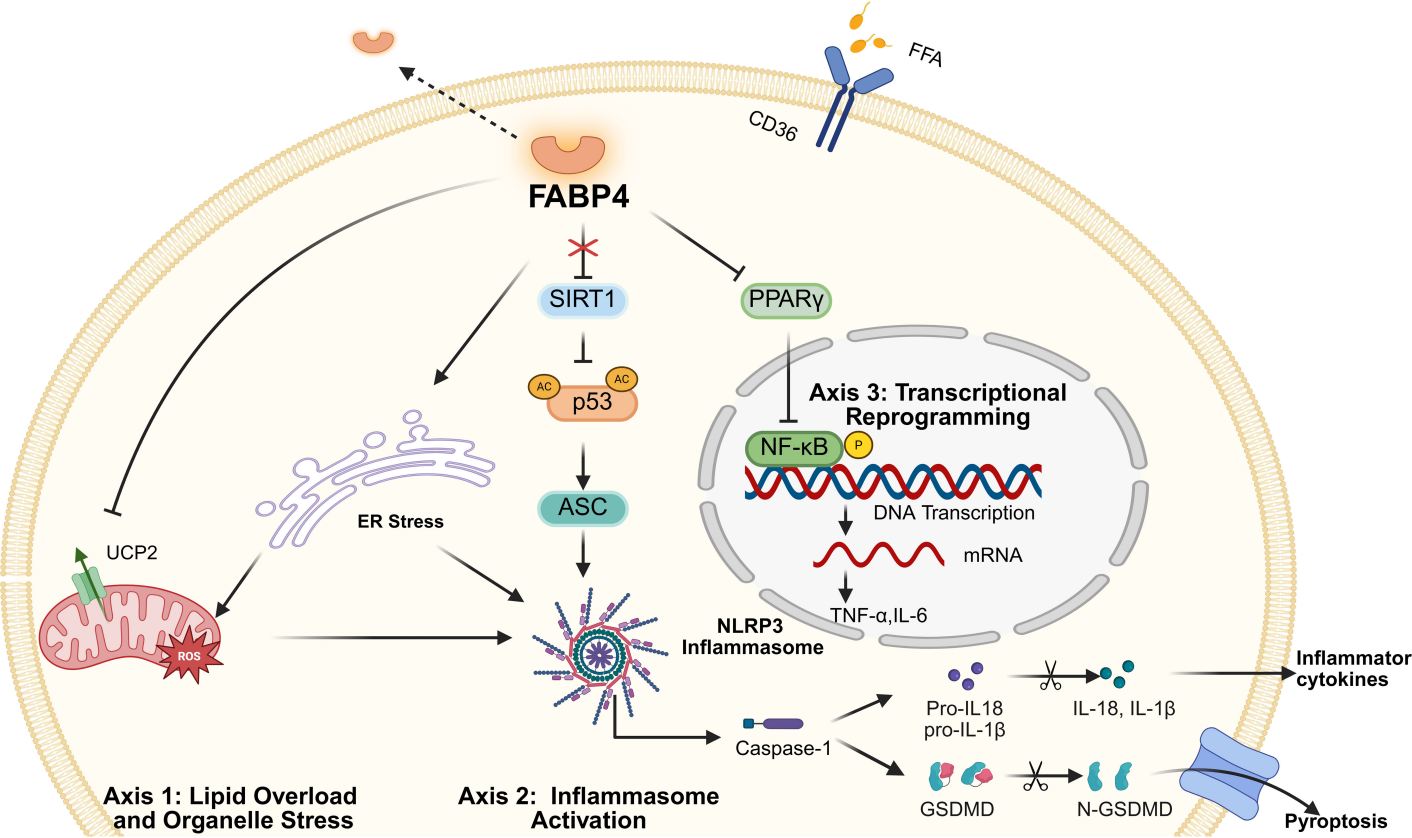
Intracellular molecular mechanisms underlying FABP4-driven immunometabolic reprogramming in macrophages. Intracellular FABP4 acts as a critical hub, translating external lipotoxicity (e.g., FFA uptake via CD36) into robust inflammatory responses through three interconnected axes. Axis 1: Lipid overload and FABP4 accumulation inhibit UCP2, triggering mitochondrial ROS overproduction and severe ER stress. Axis 2: Concurrent organelle stress, coupled with the disruption of the SIRT1–p53–ASC signaling cascade, drives NLRP3 inflammasome assembly, leading to caspase-1 cleavage and the secretion of the pro-inflammatory cytokines, IL-1β and IL-18. Axis 3: FABP4 suppresses the PPARγ pathway, unleashing nuclear translocation of NF-κB to drive robust pro-inflammatory transcriptional reprogramming. Created in BioRender. Sun H, et al. (2026) https://BioRender.com/b7kfk4t.

FABP4 influences fatty acid uptake and metabolism in immune cells via several interconnected pathways (37, 45) (**Figure 1**).

#### 2.2.1 Lipid overload and organelle stress

FABP4 facilitates intracellular lipid transport. Sustained lipid exposure elevates FABP4 expression, which suppresses autophagy and subsequently intensifies endoplasmic reticulum stress in macrophages and microglia (37, 42). FABP4 also induces significant mitochondrial dysfunction, which is a key node in immune-metabolic interactions (43). In contrast, macrophage FABP4 deficiency upregulates uncoupling protein 2 (UCP2) and reduces reactive oxygen species (ROS) production, thereby remodeling mitochondrial redox homeostasis (15), a mechanism that links lipid overload to mitochondrial dysfunction and susceptibility to iron-mediated death (44, 45).

#### 2.2.2 Inflammasome activation

FABP4 functions upstream of the NLRP3 inflammasome. Mitochondria-derived ROS surge synergizes with the mitochondrial unfolded protein response (mtUPR) to provide the necessary signals for inflammatory vesicle assembly (15). In macrophages, beyond the classical activation routes, FABP4 couples lipid metabolic disturbances to pyroptosis via the SIRT1–p53–ASC signaling cascade. Inhibition or deletion of FABP4 activates SIRT1, which inactivates p53 through deacetylation and, in turn, inhibits ASC expression, ultimately blocking NLRP3 inflammasome-mediated cellular pyroptosis (20). FABP4 expression promotes inflammasome activation by the opposite mechanism.

#### 2.2.3 Transcriptional reprogramming

Organelle stress coordinates transcriptional changes. In monocytes and macrophages, FABP4 interacts with PPARγ to form a regulatory network (39, 46), affecting the balance between lipid metabolism and inflammatory responses (18, 47). High expression of FABP4 inhibits PPARγ activity, promotes nuclear translocation of NF-κB, and drives the expression of proinflammatory cytokines; in contrast, inhibition of FABP4 restores PPARγ function and inhibits NF-κB activation, thereby establishing an anti-inflammatory regulatory loop in foamy macrophages (47).

### 2.3 FABP4 as an immunometabolic hub

By connecting lipid dysregulation to endoplasmic reticulum stress, ROS generation, NF-κB activation, and inflammasome-driven pyroptosis, these cellular pathways converge to position FABP4 as an immunometabolic hub orchestrating immunometabolic crosstalk, extending its function beyond lipid transport (30). This positioning facilitates the shift from systemic metabolic disturbance to localized innate immune activation (30). This multiple regulatory mechanism is involved in a variety of pathological processes, including promoting obesity-associated cancer progression (19), exacerbating cardiomyocyte apoptosis (48), and driving fatty acid oxidation in macrophages (49). While these mechanisms are derived primarily from non-pregnancy models, their conserved functional architecture linking lipid overload to innate immune activation provides a mechanistically plausible framework for understanding PE pathogenesis, pending direct validation in pregnancy-specific systems. If this immune reprogramming is conserved at the maternal-fetal interface, it would be expected to break down tissue-specific immune tolerance and causes severe disruption in the maternal-fetal interface (46, 50).

## 3 Spatio-temporal immunometabolic role of FABP4 in preeclampsia

### 3.1 Disruption of immune tolerance at the maternal-fetal interface

Unlike the intracellular signaling pathways derived from non-pregnancy disease models, the aberrant FABP4 expression at the maternal-fetal interface in PE represents direct clinical and histological evidence. Single-cell and spatial transcriptomic atlases of the preeclamptic placenta have revealed profound lipid metabolic reprogramming and cellular heterogeneity across distinct immune and trophoblast subsets (33, 51–53). Specifically, extravillous trophoblasts (EVTs) exhibit significant subtype-specific dysfunction in PE, with altered invasion, immunity, and stress-response pathways (51, 52), while spatial metabolomics has identified glycerophospholipid and sphingolipid metabolic disruption in trophoblast differentiation trajectories (54). However, direct single-cell quantification of FABP4 across decidual macrophage subpopulations, EVT subsets, and Hofbauer cells remains limited. Notably, in a recent single-cell atlas of the preeclamptic placenta (51), FABP4 was not identified as a significantly differentially expressed gene within the EVT compartment, suggesting that its upregulation in PE may be more pronounced in other placental populations (e.g., decidual cells or villous stroma) rather than in EVTs themselves. This important caveat underscores that the proposed role of FABP4 in EVT biology is inferred from trophoblast cell-line studies, not yet confirmed by single-cell transcriptomics. Converging evidence from histological and cell-type-specific studies permits reasoned inference: FABP4 is significantly upregulated in preeclamptic placental tissues (55–57), and metabolic stress induces FABP4 in placental trophoblast models: hyperandrogenism upregulates FABP4-mediated lipid accumulation in murine placenta (55), pharmacological inhibition or siRNA-mediated knockdown of FABP4 in HTR-8/SVneo trophoblast cells impairs mitochondrial membrane potential and ATP synthesis, mimicking the mitochondrial dysfunction observed under ischemia-hypoxia conditions in PE (28). However, direct evidence that these specific stresses operate identically in primary human EVTs remains limited. EVTs subjected to metabolic stress do not exist in isolation but interact closely with neighboring decidual macrophages (dMφ). The downstream consequences include placental lipid accumulation and lipotoxicity (55, 58), although the cell-type-specific contribution of EVTs versus other placental populations awaits finer resolution. By analogy to non-pregnancy metabolic disease models, in which FABP4 expression correlates with proinflammatory macrophage polarization (15, 18, 39), a similar association is inferred for decidual macrophages in PE, though direct evidence remains to be established. The specific cell-subset distribution of FABP4 at the maternal-fetal interface particularly its relative enrichment in EVTs versus dMφ versus Hofbauer cells awaits direct single-cell proteomic or transcriptomic validation.

This inferred localization, grounded in histological and cell-line evidence rather than single-cell quantification, establishes the working framework for the spatiotemporal cascade: FABP4-driven immunometabolic dysregulation initiates locally at the maternal-fetal interface before propagating systemically.

Immunometabolic dysfunction driven by FABP4 is particularly pronounced at the maternal-fetal interface, which is the key site of PE pathogenesis (59). During pregnancy, this microenvironment is subjected to metabolic stress from localized hypoxia and hyperlipidemia. Single-cell and spatial transcriptomics studies have shown that these stressors significantly alter the metabolic profile of placenta-resident cells and present cellular heterogeneity in both early- and late-onset PE (51–53). Histologically, FABP4 is significantly upregulated in preeclamptic placental tissues (55–57). *In vitro* studies using HTR-8/SVneo trophoblast cells demonstrate that FABP4 sustains intracellular free fatty acid homeostasis, mitochondrial membrane potential, and ATP synthesis while restraining ROS production; pharmacological inhibition or siRNA-mediated knockdown disrupts this metabolic-oxidative balance and impairs trophoblast proliferative capacity (28). These *in vitro* findings position FABP4 as a viability factor for trophoblast-like cells, yet EVT subjected to metabolic stress do not exist in isolation but interacts closely with neighboring decidual macrophages (dMφ). In normal pregnancies, dMφ maintain a tolerogenic M2 phenotype for tissue remodeling (50, 60). However, in the pathological microenvironment of PE, the activation of macrophage proinflammatory pathways is synergistically regulated by both intracellular metabolic disturbances and aberrant intercellular communication. Maternal lipid disturbances, characterized by obesity or insulin resistance, promote the accumulation of circulating saturated free fatty acids, particularly palmitic acid, at the maternal-fetal interface. Experimental models have demonstrated that localized lipotoxicity triggers inflammasome activation and inflammatory responses in placental macrophages (61). This pregnancy-specific metabolic stress constitutes an endogenous driver of abnormal FABP4 transcription and accumulation within dMφ.

Concurrently, deterioration of the local microenvironment initiates deleterious intercellular communication. Under hypoxic and stressful conditions, damaged EVT not only release hazard-associated molecular patterns (e.g., HMGB1) outwardly (24) but also secrete pathogenic EVs. EVT-derived vesicles can be taken up by neighboring dMφ, carrying specific molecules (e.g., miR-141-3p) that directly interfere with the macrophage transcriptional network and drive dMφ polarization toward proinflammatory M1-type (62–64). Whether these EVs also deliver FABP4 protein to recipient macrophages, thereby amplifying the local immunometabolic signal, is currently unknown. Nonetheless, regardless of the specific delivery mechanism, dMφ in PE exhibit significantly elevated FABP4 levels, which would be expected if the non-pregnancy macrophage mechanism is conserved to trigger their differentiation into a destructive M1-type phenotype. Trophoblast-derived extracellular vesicles enriched with miR-141-3p drive dMφ toward M1 polarization in PE (63), while other EV cargoes (e.g., miR-150-3p) additionally impair endothelial function (62), and placental macrophages exhibit distinct proinflammatory effector functions depending on clinical onset (65). The intercellular axis injured EVTs → pathogenic EV secretion → dMφ uptake and M1 polarization constitutes the core spatiotemporal cascade linking local lipotoxicity to immune dysregulation. Within this cascade, FABP4 accumulation within activated dMφ likely amplifies the inflammatory response, as inferred from macrophage studies in non-pregnancy models (15, 19); direct demonstration that EV uptake upregulates FABP4 specifically within dMφ in PE remains to be established.

Impaired mitochondrial autophagy (66) and excessive placental ferroptosis (45, 67, 68), caused by mitochondrial dysfunction (69), further exacerbate vascular injury. These inferred FABP4-driven organelle stress and death mechanisms linking lipid overload to mitochondrial dysfunction, impaired mitophagy, and ferroptosis, are hypothesized to constitute the terminal effectors of local immune-metabolic injury at the maternal-fetal interface (66–69). Ultimately, impaired remodeling of the spiral arteries and perpetuates the vicious cycle of ischemia and inflammation.

### 3.2 Systemic consequences and maternal endothelial dysfunction

**Figure 2:**
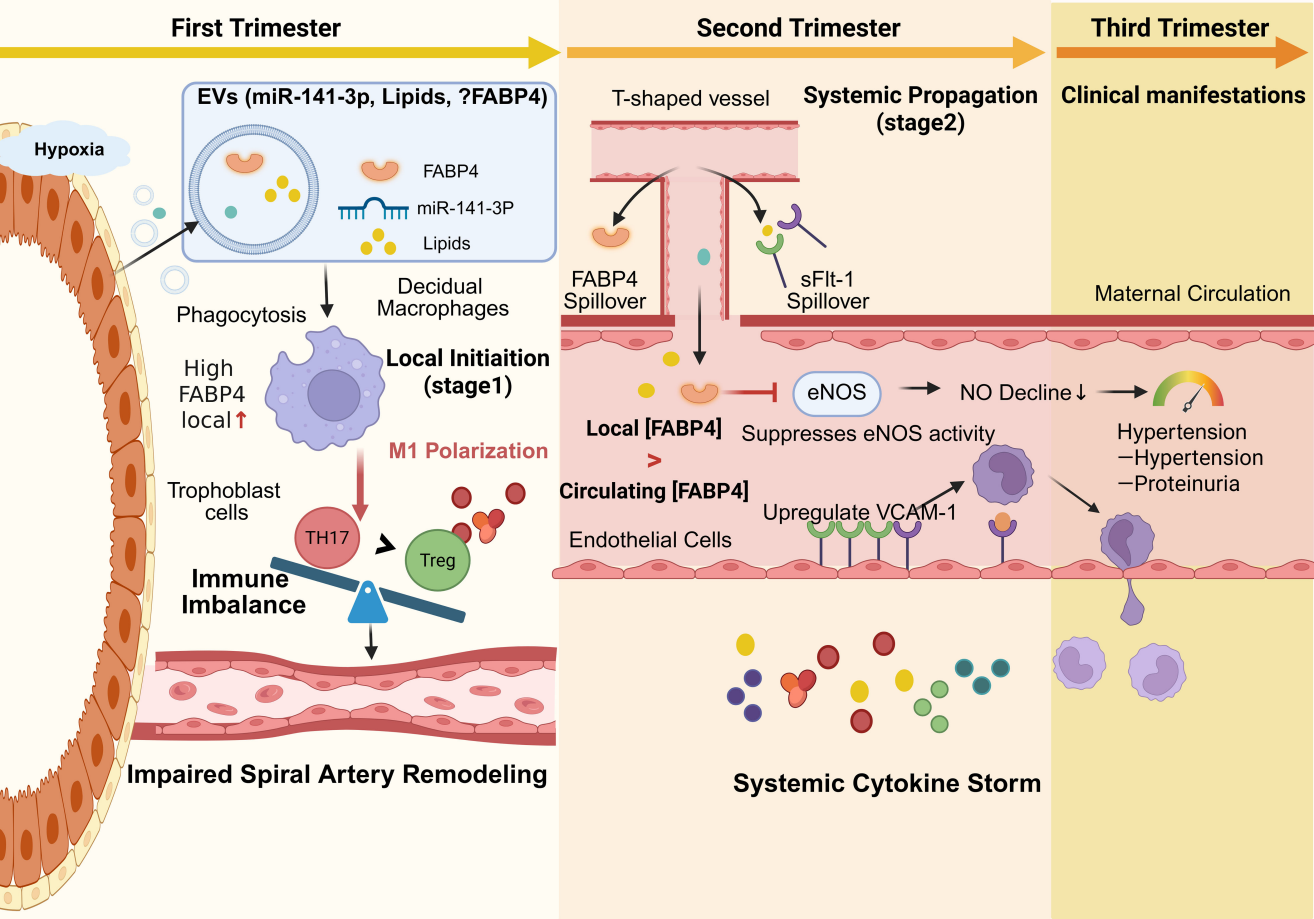
Spatiotemporal immunometabolic cascade of FABP4 from the maternal-fetal interface to systemic maternal circulation. Left panel: In the hypoxic placental microenvironment, stressed trophoblasts secrete pathogenic extracellular vesicles (EVs) carrying lipids, specific microRNAs (e.g., miR-141-3p), and potentially FABP4, although the loading of FABP4 into PE-derived trophoblast EVs remains to be quantified. Phagocytosis of these EVs by dMφ triggers profound M1 polarization, inducing an immune imbalance (Th17 > Treg) that ultimately impairs spiral artery remodeling. Right panel: Systemic spillover of FABP4 and anti-angiogenic factors (e.g., sFlt-1) into maternal circulation causes widespread endothelial dysfunction. Exogenous FABP4 suppresses endothelial nitric oxide synthase (eNOS) activity, leading to decreased NO bioavailability and uncontrolled hypertension. Concurrently, upregulated adhesion molecules (e.g., VCAM-1) on endothelial cells promote monocyte infiltration, fueling a systemic cytokine storm. Created in BioRender. Sun H, et al. (2026) https://BioRender.com/m536dl5.

Temporal hierarchy of FABP4-driven PE progression. Prospective clinical cohorts have established a clear temporal sequence in maternal circulation: serum FABP4 levels become abnormally elevated as early as the first trimester (70, 71), with further amplification in the second trimester (72), preceding the clinical onset of hypertension and proteinuria by weeks to months. Within the maternal-fetal interface, this circulating surge is hypothesized to follow local EVT and dMφ FABP4 upregulation driven by early placental metabolic stress (55–58), although direct longitudinal quantification of tissue-localized versus circulating FABP4 in the same patients remains unavailable (**Figure 2**).

Immunometabolic disturbances at the maternal-fetal interface propagate beyond the local environment. The release of pathological mediators into maternal circulation precipitates systemic vascular dysfunction. Circulating FABP4 in pregnancy appears to derive from two primary sources: first, the maternal source, namely, pregnant women with PE who are comorbid with metabolic syndrome or obesity, whose hyperactivated peripheral adipose tissue constitutes a high level of metabolic background for circulating FABP4 (29). Second, placental origin: Consistent with the classic “two-stage” model of PE (73), ischemia and lipotoxic injury may lead to necrosis or apoptosis of placental trophoblast cells, potentially releasing intracellular FABP4 into the bloodstream. Whether this occurs via passive leakage from dying cells or active non-classical secretion from stressed trophoblasts, and what quantitative contribution it makes to circulating FABP4, remains unknown in PE. Angiogenic factors (e.g., sFlt-1 and soluble endoglin (sEng)) are released concomitantly with this process (59, 74, 75) and synergistically impair endothelial function, but are not the source of FABP4.

Mechanistic convergence without established statistical correlation. While no published cohort has simultaneously performed longitudinal statistical correlation of FABP4 with sFlt-1, PlGF, and endothelial function markers (e.g., eNOS activity, soluble adhesion molecules) within the same patients during PE progression, the mechanistic convergence of these pathways is supported by independent lines of evidence: FABP4 upregulates endothelial adhesion molecules and promotes peripheral monocyte infiltration (22, 23), suppresses VEGF signaling and endothelial proliferation (76), and induces endoplasmic reticulum stress that synergizes with sFlt-1/sEng to inhibit eNOS activation (37, 39, 74, 75). An integrated biomarker panel simultaneously tracking FABP4, angiogenic factors, and endothelial function markers across gestational weeks represents a critical unmet need for future prospective studies. This propagation of inflammation from the maternal–fetal interface to the systemic circulation involves more than free molecular diffusion; intercellular communication, particularly via extracellular vesicles, contributes substantially to systemic spread. A large number of small EVs originating from the damaged placenta carry perturbed immune signals (e.g., aberrant Th17/Treg cytokine profiles) into the maternal peripheral blood (77). These placental-derived pathogenic vesicles synergize with high circulating concentrations of free FABP4 on the maternal endothelial surface.

Under conditions combining systemic lipid metabolic stress, characterized by elevated FABP4 levels, distal immune disturbances mediated by pathogenic EVs, and elevated sFlt-1 levels, the functional homeostasis of vascular endothelial cells is imbalanced. Exogenous FABP4 transcends its role as an intracellular chaperone protein and becomes an adipokine and mediator of endothelial damage with significant biological activity (14). It not only interferes with endothelial cell proliferation by modulating the vascular endothelial growth factor (VEGF)signaling pathway (76) but also FABP4-mediated endoplasmic reticulum stress, and impaired autophagy can further exacerbate the damage under conditions of lipid overload (37, 78). This organelle stress, together with sFlt-1/sEng, inhibits endothelial-type nitric oxide synthase (eNOS) activation, leading to decreased nitric oxide bioavailability, which triggers PE-specific systemic vasoconstriction and uncontrolled hypertension (59, 74, 75).

In addition, circulating FABP4 mediates bioactive lipid signaling and lactate metabolism, further exacerbating systemic inflammation (9, 49, 79). A compromised endothelial barrier promotes peripheral monocyte aggregation, adhesion, and transendothelial migration. These infiltrating monocytes differentiate into pro-inflammatory macrophages in the local microenvironment, amplifying the cytokine storm through FABP4-dependent pyroptosis pathways (20, 38). Currently, liquid biopsy techniques have detected placental-derived exosomes in maternal plasma with proteomic profiles predictive of PE occurrence (80, 81), suggesting a pathological process of the systemic spread of placental-derived inflammatory factors. Ultimately, the FABP4-driven systemic endothelial inflammatory cascade causes extensive target organ damage, suggesting that PE is an endothelial cell disease driven by immunometabolic disorders (5, 59, 73).

## 4 Clinical significance and immunometabolically targeted therapy

Given the critical role of FABP4 in PE immunometabolic dysregulation, its clinical translational potential as an early diagnostic marker for metabolically high-risk PE subtypes deserves further in-depth exploration. However, FABP4 is not proposed as a universal screening marker for all PE subtypes; rather, its greatest predictive utility lies in populations with significant dyslipidemia, insulin resistance, or metabolic comorbidities, where immunometabolic disturbance constitutes a dominant pathophysiological driver.

### 4.1 Clinical potential of immunometabolic biomarkers

The sFlt-1/PlGF ratio improves the prediction of PE in singleton pregnancies (4) and has received FDA approval and ACOG guideline endorsement for risk stratification of hospitalized hypertensive patients at 23–35 weeks (82). Nevertheless, this marker primarily reflects established placental ischemia and endothelial injury, offering limited insight into upstream immunometabolic disturbances (2, 4). In contrast, FABP4 is involved in the early stages of immunometabolic regulation and regulates the conversion of metabolic stress to the inflammatory cascade; thus, FABP4 represents a candidate biomarker for early PE detection. Prospective cohort studies have shown that elevated maternal serum FABP4 levels in early pregnancy precede classical clinical manifestations, such as hypertension and proteinuria (26, 70, 72). This elevation is particularly prominent in high-risk populations with comorbid metabolic diseases, such as gestational diabetes mellitus, and FABP4 has emerged as an independent strong predictor of PE in this population (72). In the general obstetric population, first-trimester FABP4 elevation also demonstrates independent predictive value (26). Studies in women with type 1 diabetes mellitus have further validated its predictive value: elevated levels of FABP4 in early- and mid-pregnancy are independently predictive of PE, and when incorporated into traditional clinical risk models, the net reclassification index (NRI) and the integrated discriminant improvement index (IDI) were significantly higher (71).4.1.1 Subgroup Analysis and Subtype Specificity.

PE is a highly heterogeneous syndrome, necessitating stratification of biomarkers across distinct clinical subtypes to maximize translational value. Current clinical cohorts (**Table 1**) reveal a striking pattern: FABP4’s predictive utility is most pronounced in metabolically high-risk subgroups.

**Table 1:** Summary of clinical evidence linking FABP4 to preeclampsia: stratification by clinical subtypes and metabolic comorbidities.

Studies	Study design	Population (sample size)	PE subtype	Metabolic comorbidity	FABP4 type	Detection timing	Key findings
Scifres et al. (26)	Prospective nested case-control	Pregnant women (n=67 cases, 134 controls)	Not Specified	Not Specified	Serum	1st (8–13 wks) and 2nd (24–28 wks) trimester	Elevated serum FABP4 prior to PE onset; independently predictive after adjusting for BMI and BP
Wotherspoon et al. (71)	Prospective cohort	Pregnant women with T1DM (n=710)	Not Specified	T1DM	Serum	1st and 2nd trimester	Increased serum FABP4 prior to PE onset; improves NRI and IDI.
Li et al. (72)	Nested case-control	GDM women (n=82 matched)	Not Specified	GDM	Plasma	2nd trimester (24–32 weeks)	Increased plasma FABP4 independently predicts GH/PE in GDM.
Paiboonborirak and Phupong (70)	Prospective cohort	Singleton pregnancies (n=330)	Not Specified	Not Specified	Serum	1st trimester (11–13^+6^ weeks)	Serum FABP4 + UtA-PI yields high sensitivity and NPV for PE prediction.
Yan et al. (56)	Case-control	PE patients vs. controls (n=58)	Mixed (mild/severe; not stratified by onset)	Not Specified	Placental tissue	Placental tissue (at delivery)	Increased placental FABP4 associated with trophoblast dysfunction.

PE, preeclampsia; EOPE, early-onset preeclampsia; T1DM, type 1 diabetes mellitus; GDM, gestational diabetes mellitus; NRI, net reclassification improvement; IDI, integrated discrimination improvement; UtA-PI, uterine artery pulsatility index; NPV, negative predictive value, “Not Specified” indicates that the original clinical study did not provide explicit stratification or recruitment criteria based on the respective clinical parameter (e.g., timing of PE onset or pre-existing metabolic comorbidities).

In women with gestational diabetes mellitus, second-trimester plasma FABP4 independently predicts subsequent gestational hypertension and PE (72), suggesting that pre-existing insulin resistance and lipotoxic stress amplify FABP4’s immunometabolic signal. Similarly, in type 1 diabetes mellitus pregnancies, first- and second-trimester serum FABP4 elevation precedes PE onset and improves net reclassification indices (71), indicating that chronic dyslipidemia sensitizes the maternal-fetal interface to FABP4-driven pathological reprogramming. Conversely, in metabolically normal singleton pregnancies (70), FABP4 combined with uterine artery Doppler achieves high sensitivity, but the incremental predictive gain over conventional angiogenic markers appears more modest.

Stratification by clinical onset specifically early-onset (<34 weeks) versus late-onset PE remains critically underexplored. As highlighted in **Table 1**, most current studies do not specify onset timing within their FABP4 analyses. This represents a significant resolution gap: EOPE, frequently characterized by profound placental dysfunction and inadequate spiral artery remodeling, may exhibit distinct FABP4 spatial patterns compared to LOPE, which more commonly arises in the context of pre-existing maternal metabolic vulnerability.

Therefore, based on current stratified evidence, FABP4 is more suitable as an early biomarker for metabolism-associated high-risk PE rather than a universal marker for all PE subtypes. Future prospective cohorts must prioritize detailed stratification by both metabolic background and onset timing to fully delineate the distinct immunometabolic profiles across different PE phenotypes.

Complementing these stratified serum findings, liquid biopsy techniques have identified maternal plasma exosomes and soluble protein profiles capable of predicting and molecularly typing early-onset PE (80). For example, quantitative proteomic analysis of serum-purified exosomes has revealed differential regulation of angiogenic and inflammatory networks in EOPE (81). Plasma small- and medium-sized exosomes and Th17/Treg cytokine profiles reflect immune imbalances at the maternal-fetal interface (77) and correlate with specific placental miRNAs (e.g., miR-210 and miR-148a/152) (83), providing a new means of assessing placental toxicology and endocrine disruption (84) Although these exosome-based diagnostic methods have laid some groundwork, the specific quantification of FABP4 in circulating exosomes remains to be clinically validated.

Combining early pregnancy serum FABP4 levels with the uterine artery Doppler pulsatility index is expected to construct a highly sensitive multidimensional diagnostic model (70), which can further enhance the predictive efficacy if combined with the conventional sFlt-1/PlGF ratio (4, 82). Mendelian randomization studies linking multidimensional plasma lipid profiles to PE causality also support this comprehensive strategy (7). By simultaneously capturing the dimensions of “impaired angiogenesis” and “immunometabolic disorders”, this integrated approach can significantly improve the predictive specificity of early-onset and severe PE.

### 4.2 Intervention strategies based on the FABP4 network of mechanisms

These mechanisms implicate FABP4-mediated immunometabolic reprogramming in the pathogenesis of PE. Therefore, intervening in this metabolic-inflammatory axis to restore maternal-fetal immune tolerance may be a potential therapeutic strategy. However, the pharmacological specificity of the drug must be evaluated separately from fetal safety during its translation to the obstetrics clinic (**Table 2**).

**Table 2:** Categorization and translational potential of intervention strategies targeting the FABP4 immunometabolic axis.

Studies	Intervention/Agent	Target cells & disease model	Main immunometabolic mechanisms	Key outcomes
Panel A - Preclinical Pharmacological Probes				
Sun F et al. (48)	BMS309403 (Specific inhibitor)	Cardiomyocytes (Hypoxia-induced)	Decreased ER stress	Protects against cardiomyocyte apoptosis
Zhu et al. (85)	BMS309403 (Specific inhibitor)	Cardiac fibroblasts/Macrophages (Ang II-induced)	Inhibits NLRP3 inflammasome activation	Reduces cardiac fibrosis and remodeling
Sun Z et al. (86)	BMS309403 (Specific inhibitor)	Pregnant mice (Systemic administration)	Disrupts systemic maternal-fetal lipid transport	Fetal toxicity alters fetal brain lipid profiles and causes autism-like neurodevelopmental disorders
Zhang et al. (47)	Oridonin (Natural compound)	Macrophages (Atherosclerosis)	Inhibits FABP4/Enhances PPARγ signaling	Inhibits foam macrophage formation and systemic inflammation
Ruan et al. (78)	FOS upregulation/FABP4 knockdown	HUVECs (Palmitic acid-induced endothelial dysfunction model)	Inhibits the ERK/STAT-1 signaling pathway	Repairs endothelial cell dysfunction and mitigates apoptosis
Panel B - Pleiotropic Metabolic Modulators for Repurposing				
Feng et al. (87)	Metformin (Repurposed drug, preclinical)	Macrophages (Metabolic disease)	AMPK-independent FOXO1 nuclear exclusion, suppressing FOXO1-driven FABP4 transcription	Reverses macrophage dysfunction and prevents atherogenesis
Cluver et al. (88, 89)	Metformin (Repurposed drug, clinical)	Pregnant women (Preterm PE trial)	Enhances systemic immunometabolic modulation	Safely prolongs gestation by ~7.6 days (PI 2 trial, n=180); PI 3 confirmatory trial ongoing (n=500)
Furuhashi et al. (90)	Sitagliptin (DPP-4 Inhibitor)	Type 2 Diabetes patients	DPP-4 inhibition	Decreased circulating serum FABP4 concentrations; clinically dampens systemic metabolic inflammation markers
Redondo-Delgado et al. (11)	Exercise training (Lifestyle)	Pregnant women (Systemic profile)	Systemic regulation of lipid metabolism and oxidative stress	Modulates maternal biomarkers and blunts pathological inflammation
Panel C - Future Targeted Delivery Platforms				
Safford et al. (92)	Lipid Nanoparticles (LNPs)	Placenta (Maternal-fetal interface)	Enables localized delivery of therapeutic mRNA	Potential targeted delivery system to maximize maternal therapy and prevent fetal toxicity

ER, endoplasmic reticulum; Ang II, angiotensin II; HUVECs, human umbilical vein endothelial cells; PPARγ, peroxisome proliferator-activated receptor gamma; ERK, extracellular signal-regulated kinase; STAT-1, signal transducer and activator of transcription 1; AMPK, AMP-activated protein kinase; PE, preeclampsia; PI 2, Preeclampsia Intervention 2 trial; DPP-4, dipeptidyl peptidase-4; LNPs, lipid nanoparticles; FOXO1, Forkhead box O1.

Pharmacological Probe Value versus Translational Barriers of Direct-Targeted Inhibitors: Small-molecule inhibitors (e.g., BMS309403) can achieve precise direct-targeted inhibition by competitively binding to the lipid-binding pocket of FABP4 and have demonstrated significant efficacy for cardiovascular protection in specific *in vitro* models and in non-pregnant animals (48, 85). However, BMS309403 is currently a pharmacological probe compound only, with no clinically translatable therapeutic window in human pregnancy. As a small lipophilic molecule (molecular weight ~400 Da), it is predicted to cross the placental barrier via passive diffusion, leading to unrestricted fetal exposure. Preclinical evidence indicates that systemic inhibition of FABP4 during gestation disrupts maternal-fetal lipid transport and alters fetal brain lipid profiles, resulting in autism-like neurodevelopmental abnormalities in offspring (86). Consequently, no safe gestational window exists for systemic administration, because fetal neurodevelopment relies on precisely regulated lipid metabolism across all trimesters. These agents therefore serve exclusively as experimental tools to validate FABP4-driven pathogenic mechanisms (48, 85), and their clinical application in obstetrics is precluded until placental-impermeable derivatives or strictly localized delivery systems become available.

**Figure 3:**
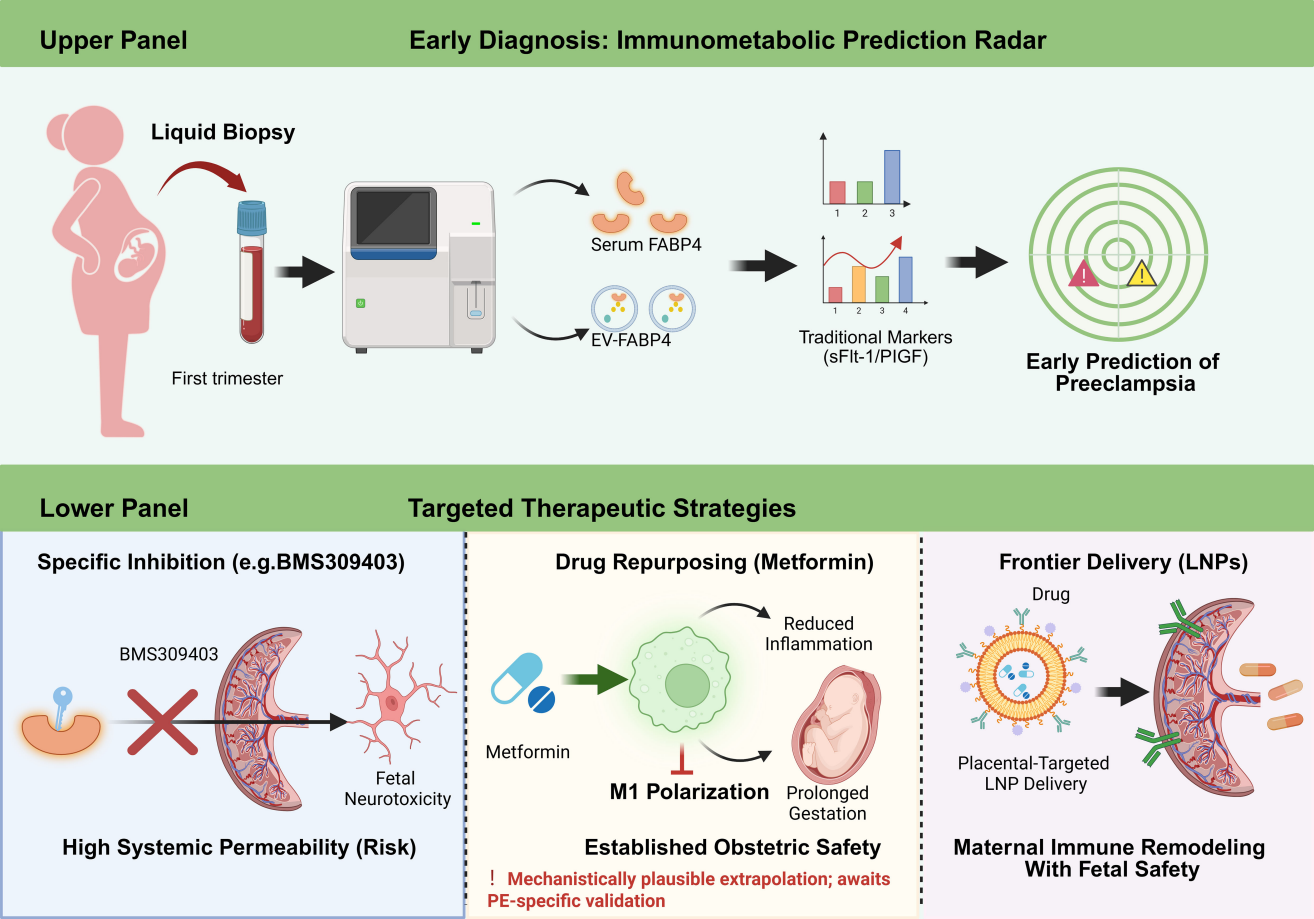
Clinical translation: Early immunometabolic prediction and targeted therapeutic strategies for preeclampsia. Upper panel: An “immunometabolic prediction radar” integrating first-trimester liquid biopsies. Detecting elevated circulating serum FABP4 and EV-encapsulated FABP4, alongside traditional angiogenic markers (sFlt-1/PlGF), significantly enhances the early prediction of preeclampsia. Lower panel: Potential targeted therapeutic strategies and associated clinical challenges. While specific FABP4 inhibitors (e.g., BMS309403) effectively block inflammation, their high systemic permeability poses a risk of fetal neurotoxicity. Repurposing established drugs, such as metformin, offers obstetric safety and safely prolongs gestation by reducing inflammation and reversing destructive macrophage polarization. Frontier placental-targeted delivery systems, such as lipid nanoparticles (LNPs), conceptually adapted from placental mRNA delivery platforms represent a speculative but highly promising avenue to achieve robust maternal immune remodeling while meticulously safeguarding fetal health, pending rigorous preclinical safety validation. Created in BioRender. Sun H, et al. (2026) https://BioRender.com/yk65tug.

Indirect modulation and repurposing of pleiotropic metabolic modulators: Given that new drug development in pregnancy is limited by extremely stringent safety guidelines, screening pleiotropic metabolic modulators with an established obstetric safety record for drug repurposing is a more viable translational strategy. Metformin typifies this indirect strategy. Although not a direct FABP4 ligand, metformin indirectly ameliorates FABP4-mediated lipid accumulation and macrophage dysfunction via an AMPK-independent pathway, specifically by suppressing FOXO1-driven FABP4 expression (87). It is critical to emphasize that this AMPK-independent FOXO1–FABP4 axis has been established exclusively in non-pregnant metabolic disease and atherosclerosis models (87), not in pregnancy-specific systems. In the context of PE, we advance this axis strictly as a mechanistically plausible extrapolation: reduced FABP4 expression would theoretically dampen M1 macrophage polarization and NLRP3 inflammasome activation at the maternal-fetal interface, thereby potentially alleviating local inflammation and promoting spiral artery remodeling (**Figure 3**). Direct validation in PE-specific decidual macrophages, trophoblasts, or human placental organoids is entirely lacking and represents an urgent research priority. A recent double-blind randomized trial (PI 2 trial) demonstrated that extended-release metformin safely prolonged pregnancy by approximately 7.6 days in pregnant women with early-onset PE who were receiving expectant therapy (88); its clinical efficacy will be further validated in the ongoing phase III PI 3 trial (89). In addition, dipeptidyl peptidase-4 (DPP-4) inhibitors (e.g., sitagliptin) have been found to significantly downregulate circulating FABP4 levels as a downstream effect while ameliorating the systemic inflammatory background in patients with metabolic syndrome (90). Large international cohort studies provide preliminary indications of their relative safety in periconceptional exposure (91); however, their potential immunometabolic modulatory role in PE prevention remains to be specifically evaluated.

Lifestyle interventions and targeted delivery systems: Non-pharmacological interventions remain the foundation of immunometabolic management. Studies have shown that scientific dietary management combined with aerobic exercise during pregnancy can effectively reduce maternal lipid overload and significantly downregulate FABP4 expression and maternal inflammatory markers as a result of systemic metabolic improvement (11).

Translational Implications of Systemic versus Local Administration: The foregoing analysis underscores a fundamental pharmacological distinction between systemic and local administration paradigms. Systemic administration of direct FABP4 inhibitors whether by oral or parenteral routes carries an inevitable risk of placental transfer and fetal exposure, thereby negating any conceivable therapeutic index in pregnancy. By contrast, localized delivery strategies, exemplified by placenta-targeted lipid nanoparticles (LNPs) engineered for maternal-fetal interface retention (92), offer a conceptually distinct translational avenue. Such platforms, originally developed for placental mRNA delivery (92), are theoretically designed to penetrate maternal vasculature while achieving localized tissue retention, potentially minimizing fetal exposure, although their application to FABP4-targeted immunometabolic modulation in PE remains purely conceptual. Their placental permeability, biodistribution kinetics, and dose-toxicity relationships in the context of immune-modulatory cargo remain entirely uncharacterized in human pregnancy and require rigorous preclinical evaluation before clinical translation can be contemplated (**Figure 3**).

## 5 Discussion

This study integrated clinical evidence on PE (26, 56, 57, 70–72, 93) with cross-disease mechanistic studies (13, 15, 18–20, 39, 40) to propose a theoretical framework positioning FABP4 as an immunometabolic hub connecting metabolic disorders and immune dysregulation. The construction of this theoretical framework relies on two major indirect pieces of evidence: (1) correlation analysis between abnormal FABP4 expression and clinical phenotypes in patients with PE, and(2) pathologic extrapolation based on FABP4 function in non-pregnant metabolic disease models.

### 5.1 Limitations and future directions of mechanistic studies

Future studies might address these gaps through several approaches.

#### 5.1.1 Single-cell resolution

Although single-cell RNA sequencing initially characterized the molecular profile of preeclamptic trophoblast cells and the immune microenvironment (33, 51, 53), there is a lack of fine-grained characterization of the dynamic evolution of FABP4 in specific subpopulations, such as dMφ. Future studies should incorporate multicolor flow cytometric analysis to sort macrophage subpopulations with high FABP4 expression from pathological placental tissues and detect the activation of the local inflammatory cascade at the transcriptional and protein levels using quantitative PCR and ELISA. This validation model, which combines histology and classical immunology, will provide key evidence for establishing cell-specific functions of FABP4.

#### 5.1.2 *In situ* analysis of intercellular communication

The success of spiral artery remodeling relies on highly precise intercellular coordination (94). Future studies should extensively employ spatial transcriptomics in conjunction with multiplex immunofluorescence (36, 54) to visualize the physical contact of damaged extravillous trophoblast cells with neighboring infiltrating macrophages and the trajectory of transcellular transmission of pathologic vesicles or HMGB1 at the three-dimensional *in situ* level of the tissue (24, 67–69). This provides an indispensable morphological basis for understanding the spatiotemporal dynamics of FABP4 in the evolution of local pathology.

#### 5.1.3 Pregnancy-specific functional models

The current absence of PE-specific conditional knockout models (e.g., macrophage-specific or trophoblast-specific FABP4 knockout in pregnant mice) and human placental organoid systems represents a critical unmet need in the field. Given that the available mechanistic evidence comes mainly from non-pregnant disease models, establishing such pregnancy-specific functional systems is an urgent priority. This review explicitly highlights this gap to guide future investigation. Such models can directly validate (1) the causal role of trophoblast FABP4 expression in placental lipid accumulation and lipotoxicity (55, 56, 58); (2) the effect of FABP4 deletion in decidual macrophages on the M1/M2 polarization balance and spiral artery remodeling (50, 60, 65); and (3) the dose-effect relationship between circulating FABP4 and endothelial dysfunction (76, 78).

### 5.2 Challenges and prospects for clinical translation

Although interventions targeting FABP4 are theoretically highly promising, the core challenge lies in ensuring the safety of drugs during pregnancy when translating these findings into obstetric clinical practice. Lipid metabolism plays a critical role in early embryonic neural development, and the use of FABP4-specific inhibitors capable of crossing the placental barrier (such as BMS309403) may interfere with the normal development of the fetal nervous system (86). Consequently, such systemically active inhibitors can currently serve only as research tools to validate pathological mechanisms that is, as “pharmacological probes” (48, 85) and are not yet ready for clinical application in the near term.

Given that the development of new drugs during pregnancy must adhere to extremely stringent safety guidelines, screening for multifunctional metabolic modulators with established safety profiles in obstetrics and drug repurposing represents a more feasible strategy. As mentioned earlier, metformin (87)and dipeptidyl peptidase-4 (DPP-4) inhibitors (such as sitagliptin) (90) can indirectly improve FABP4-mediated lipid accumulation and systemic metabolic inflammation. In recent relevant clinical trials, these drugs have demonstrated preliminary potential to extend gestational duration and have relatively good safety profiles (88, 89, 91). This system-level metabolic regulation represents a pragmatic choice that balances maternal and fetal safety at this stage. From a long-term perspective, to avoid potential fetal toxicity associated with systemic medication, the development of locally targeted delivery systems capable of penetrating the maternal vasculature and precisely retaining at the maternal-fetal interface is a key future direction (92). Such systems provide an important technical platform for precision interventions based on the FABP4 mechanism.

Furthermore, given the high clinical heterogeneity of PE, future clinical trials should adopt a precision approach, focusing on high-risk pregnant women with significant dyslipidemia (e.g., those with obesity, insulin resistance, or gestational diabetes). To clarify the preventive and therapeutic value of FABP4 and its related inflammatory pathways in this specific group, more prospective cohort studies are warranted.

In summary, FABP4 not only performs the fundamental physiological function of acting as an “intracellular lipid chaperone” but also serves as a critical immunometabolic hub in the pathogenesis of preeclampsia. FABP4 contributes to driving the local inflammatory storm at the maternal-fetal interface and is closely associated with the amplification of systemic endothelial damage in the mother. Under metabolic stress, pathological lipid interactions between trophoblast cells and macrophages (55) trigger local immune dysregulation, primarily manifested as M1 polarization (15, 18) and NLRP3 inflammasome activation (15, 20), ultimately affecting spiral artery remodeling and widespread vascular pathology (5, 59). Currently, treatment strategies for PE are shifting from symptom management toward mechanistic interventions (2). Modulating the FABP4 metabolic axis presents a novel avenue for investigating the mechanisms underlying maternal-fetal immune tolerance. This hypothesis requires further validation in PE-specific *in vivo* and *in vitro* models, including conditional knockout systems and human placental organoids. Consequently, with the advent of high-resolution spatio-temporal omics (33, 53, 54) and precision local delivery technologies (92), novel intervention strategies predicated on immune-metabolic interactions have emerged as a central focus in the study of pregnancy complications.
